# PTA-Det: Point Transformer Associating Point Cloud and Image for 3D Object Detection

**DOI:** 10.3390/s23063229

**Published:** 2023-03-17

**Authors:** Rui Wan, Tianyun Zhao, Wei Zhao

**Affiliations:** 1 School of Automation, Northwestern Polytechnical University, Xi’an 710129, China; wanruinpu@mail.nwpu.edu.cn; 2 Institute of Photonics & Photon Technology, Northwest University, Xi’an 710127, China; zwbayern@nwu.edu.cn

**Keywords:** automatic driving, 3D object detection, multi-modal fusion, point cloud

## Abstract

In autonomous driving, 3D object detection based on multi-modal data has become an indispensable perceptual approach when facing complex environments around the vehicle. During multi-modal detection, LiDAR and a camera are simultaneously applied for capturing and modeling. However, due to the intrinsic discrepancies between the LiDAR point and camera image, the fusion of the data for object detection encounters a series of problems, which results in most multi-modal detection methods performing worse than LiDAR-only methods. In this investigation, we propose a method named PTA-Det to improve the performance of multi-modal detection. Accompanied by PTA-Det, a Pseudo Point Cloud Generation Network is proposed, which can represent the textural and semantic features of keypoints in the image by pseudo points. Thereafter, through a transformer-based Point Fusion Transition (PFT) module, the features of LiDAR points and pseudo points from an image can be deeply fused under a unified point-based form. The combination of these modules can overcome the main obstacle of cross-modal feature fusion and achieves a complementary and discriminative representation for proposal generation. Extensive experiments on KITTI dataset support the effectiveness of PTA-Det, achieving a mAP (mean average precision) of 77.88% on the car category with relatively few LiDAR input points.

## 1. Introduction

**Figure 1:**
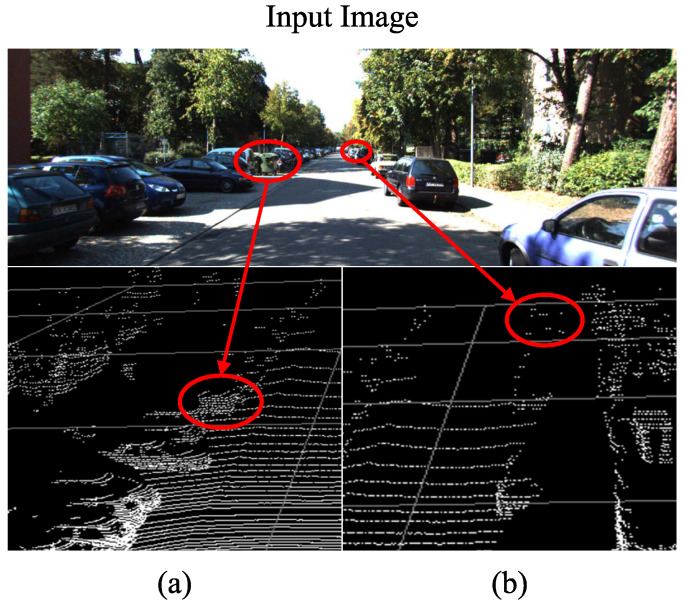
(**a**) The background obstacle has a similar 3D structure to a car. It is difficult to distinguish them in the point cloud. (**b**) The points of the distant car are too sparse to determine the bounding box.

3D object detection is a fundamental perception task for autonomous driving, which usually takes point clouds or images as input. It aims to estimate the 3D bounding boxes of objects and recognize their categories. Due to the success of convolution neural network (CNN) in 2D object detection, image-based 3D object detection has emerged to obtain spatial clues about the objects. Although the image elaborates the front-view projection of objects in a 3D scene, accurate depth measurement is still required in order to localize 3D objects. In the last decade, 3D sensors such as LiDAR have developed rapidly from the survey [1]. With these devices, researchers can obtain point clouds that reflect the relative positions of the sensor and the obstacles for 3D object detection. Early works relying on LiDAR points (e.g., PointRCNN [2] and VoxelNet [3]) have achieved superior results over image-based detection methods. However, they suffer from the poor semantic information of point clouds, as shown in Figure 1a. In addition, the objects in Figure 1b are difficult to detect using LiDAR-only methods because the distant point cloud is extremely sparse. In contrast, an image is an array of pixels and can provide continuous textural structure, which is in favor of distinguishing false-positive instances. Intuitively, it is essential to design a multi-modal 3D object detection method to exploit both the geometry clues in the point cloud and the textural clues in the image.

As can be seen from the KITTI [4] leaderboard, there is still a gap between the mean Average Precision (mAP) of multi-modal methods and LiDAR-only methods. **The first performance bottleneck encountered by multi-modal methods is the capability to extract intra-modal features.** According to the related works [5,6,7,8,9,10,11,12,13,14,15,16,17,18], normally point cloud features are extracted via PointNet++ [19]/3D sparse convolution [20], while image features are extracted through 2D convolution [21]. However, the useful long-distance dependencies in each modality are difficult to capture due to the local receptive fields of these building blocks. To balance speed and accuracy, multi-modal methods usually reduce the number of point clouds and image size of the input. The approach will cause serious long-distance information loss and reduce the detection accuracy.

**The second performance bottleneck of multi-modal methods is restricted by the fusion mode of inter-modal features.** In order to utilize mature 2D object detectors and deep learning methods for point cloud, Frustum-PointNet [7] generated 2D proposals for images and lifted them into frustums. Then it predicted the bounding box from points in the extruded frustum. As proposed in the investigation [22], using multiple data separately does not take their complementarity into account. Later, researchers attempted to fuse point cloud and image features at the high-resolution feature level. AVOD [6] as a pioneering work was proposed to project point clouds into Bird’s eye view (BEV) and aggregate BEV features and image features in anchors to generate proposals. Owing to the quantized errors during projection, the result is affected by an inaccurate alignment between the two features. Some works turned to more fine-grained multi-modal feature fusion, namely feature fusion at the point level. PointPainting [10] performed image semantic segmentation and appends the semantic score of the projected position on the image to the LiDAR points. The enhanced features are sent to a point-based method for proposal generation. Although the mAP is relatively improved, the simple feature concatenation is not enough to effectively fuse image and point cloud features. In summary, the main reason limiting their accuracy is the problematic fusion of multi-modal data.

To conquer the first performance bottleneck, we strive to learn more expressive point cloud features and image features. To extract point cloud features, the transformer [23] proposed in machine translation is used to construct the feature extraction module. Recent studies [24,25] have shown that transformer is capable of point cloud classification and segmentation. Compared with CNNs, transformer is developed based on a self-attention mechanism and can capture more distant dependencies. Therefore, relying on self-attention, Point Transition Down (PTD) and Point Transition Up (PTU) modules are designed to extract point cloud features. In contrast, the extraction of image features in multi-modal detection is still an open problem. Inspired by the application of pseudo point cloud (PPC) in 3D object detection by MDPPC [26], a Pseudo Point Cloud Generation network that converts image keypoints into PPCs is developed, and then the PPCs are used to acquire the features of image keypoints from a novel feature frustum. Attributed to the unified form of PPC and LiDAR points, both image features and point cloud features can be learned in the form of points.

To address the second performance bottleneck, a two-stream feature extraction network, based solely on transformer, is developed to solve the fusion of inter-modal features. Specifically, the two-stream structure consists of a point cloud branch and a PPC branch. The two branches independently learn high-level point cloud features and PPC features. In particular, the image features of the object keypoints are used as the initial PPC features, and these features are further analyzed and encoded in the PPC branch. Benefiting from the unified feature representation, a Point Fusion Transition (PFT) module is developed, which accurately fuses the two features at the point level to highlight the key cues across the two modalities.

In general, we present a multi-modal 3D object detection method PTA-Det, constructed on the basis of point-level feature learning and fusion. Accompanied by a series of modules, the mAP of multi-modal 3D object detection can be improved with better robustness and accuracy. Our main contributions are summarized as follows:

- The PPC generated by a Pseudo Point Cloud Generation network, a point-based representation of image feature, has been leveraged for multi-modal 3D object detection.
- A two-stream feature extraction network entirely relying on transformer has been developed, to learn intra-modal features and inter-modal features at the point level.
- Competitive results on KITTI dataset have been achieved. Results demonstrate that our model is compatible with most LiDAR-only detectors and can be easily upgraded to a multi-modal detector.

## 2. Related Work

**PPC-based 3D Object Detection.** From the investigation by Reading et al. [27], image-based detection methods show unsatisfactory results owing to lacking direct depth measurement. However, Wang et al. [28] argued that image-based methods are mainly affected by the representation of the data rather than its quality. They converted the depth image to the PPCs and applied a point-based method to detect objects. Pseudo-LiDAR [26] performed depth estimation and proposal prediction on image. For each proposal, a point cloud frustum is extracted from the PPCs obtained from the depth image transformation. Nevertheless, both of these methods ignore the depth error introduced into the PPC, which further affects their detection accuracy. To solve the problem, Pseudo-LiDAR++ [29] utilized extremely sparse LiDAR points to correct their nearby PPCs to achieve accurate depth prediction. Wang et al. [30] proposed a PnP module that integrated sparse depth values into an intermediate feature layer to correct depth prediction. In addition to depth correction, End-to-End Pseudo-LiDAR [31] jointly trained depth prediction and object detection for accurate proposals. Hence, in our proposed Pseudo Point Cloud Generation network, we not only dynamically generate PPCs using depth prediction, but also apply corresponding image features for the subsequent detection pipeline.

**Multi-modal based 3D Object Detection.** According to the different fusion strategies, the existing multi-modal detection methods can be divided into three categories: result-level, proposal-level and point-level methods. In the result-level methods [5,7,11,12,17], it is common to utilize the feature of one modality to generate the proposal, and utilize the feature of the other modality in the proposal to generate the bounding box. These methods have high recall even when the object is far or occluded, but their accuracy is limited by ignoring the complementarity between different data. The proposal-level methods [6,9,16,32,33] take the encoded features of image and point cloud as inputs, and fuse the two features in anchors to generate proposals. These methods benefit from multi-modal data and can generate high-quality proposals. However, their performance is affected by irrelevant information mixed in the anchors and inaccurate feature alignment. The point-level methods [10,13,14,15,34] have shown promising results. ImVoteNet [14] fused 2D votes of image and point cloud features in a point-wise concatenation manner, but the approach is insufficient to fuse the two features. To address the drawback, EPNet [13] proposed the LI-Fusion module that adaptively fuses point cloud features with image features according to the importance of the image feature channel. EPNet++ [15] proposed the CB-Fusion module that added the fusion direction from the point domain to the image domain. It showed that bi-direction interaction approach leads to a more comprehensive and discriminative feature representation. Recently, several transformer-based multi-modal detection methods [34,35] have been proposed. Dosovitskiy et al. [36] demonstrated that transformers have comparable expressive power in CNN. Due to the powerful feature aggregation ability of transformer, we aim to design a two-stream feature extraction backbone based solely on its attention mechanism. Unlike existing multi-modal backbones, the proposed backbone can handle both point clouds and pseudo points and capture the useful patterns about objects in each modality. A PFT module, as a submodule of the backbone, is also proposed to perform feature interactions at multiple scales to supplement the missing object information in each modality.

## 3. Method

**Figure 2:**
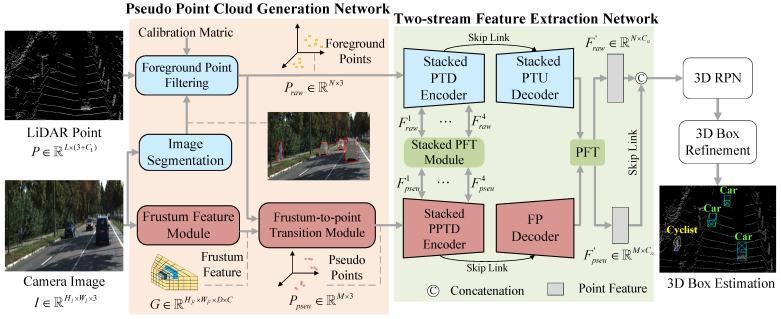
Illustration of the architecture of PTA-Det. It is composed of four parts: (1) Pseudo Point Cloud Generation Network for generating the coordinates and features of PPCs, (2) Two-stream Feature Extraction Network for learning point-based intra-modal and inter-modal features, (3) 3D RPN for proposal generation, (4) 3D Box Refinement for rectifying proposals.

In this research, we present a multi-modal 3D object detection method named PTA-Det. As shown in Figure 2, the PTA-Det mainly consisted of a Pseudo Point Cloud Generation Network, a Two-stream Feature Extraction Network, a 3D Region Proposal Network (RPN), and a 3D Box Refinement Network. The Pseudo Point Cloud Generation Network comprised a Frustum Feature module and a Frustum-to-point Transition module. The Two-stream Feature Extraction Network included a point cloud branch and a PPC branch. The former contained a stacked PTD encoder and a stacked PTU decoder, and the latter contained a stacked pseudo PTD (PPTD) encoder and a stacked FP decoder. Meanwhile, the stacked PFT module was used to connect the two branches at multiple levels. In the following, the four subnetworks utilized in the investigation were elucidated in sequence.

### 3.1. Pseudo Point Cloud Generation Network

In this network, image was transformed into PPCs which were further utilized to represent image features. During processing, the image depth was predicted in a semi-supervision manner, and LiDAR points were projected onto the image to obtain sparse depth labels, which were used to supervise depth prediction. With the help of the foreground mask from Mask-RCNN [37] and the predicted depth image, the foreground pixels can be converted into pseudo points. At the same time, adhering to CaDDN [27], the Frustum Feature Module was used to construct the frustum feature. Then, the PPC features were obtained by interpolating the frustum feature in the Frustum-to-point Transition module.

**Figure 3:**
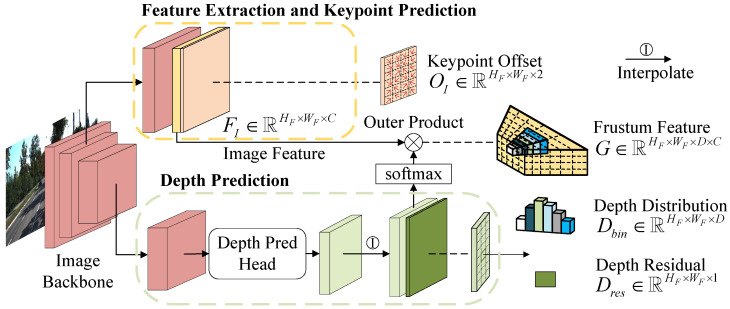
Illustration of Frustum Feature Module.

**Frustum Feature Module.** In order to make full use of the image information, a Frustum Feature module was constructed to generate frustum feature. In Figure 3, extracting image features and predicting image depth were two fundamental steps. Similar to CaDDN [27], ResNet-101 [38] was utilized as the backbone to process images and the output of its Block1 was used to collect image features $F_{I}\in R^{H_{F}\times W_{F}\times C}$, where $H_{F}$,$W_{F}$ were the height and width of the image feature, and *C* was the number of feature channels.

On the other hand, a depth prediction head was applied to the output of the image backbone to predict image depth. The depth prediction was viewed as a bin-based classification problem and the depth range was discretized into D bins by the discretization strategy LID [39]. Then the depth distribution $D_{bin}\in R^{H_{F}\times W_{F}\times D}$ and depth residual $D_{res}\in R^{H_{F}\times W_{F}\times 1}$ can be obtained.

Early depth estimators [27,39,40] computed the loss over the entire image including a large number of background pixels. These methods placed over-emphasis on background regions in depth prediction. According to Qian et al. [31], background pixels can occupy about 90% of all pixels in the KITTI dataset. Therefore, instead of calculating the loss of all image pixels, the off-the-shelf image segmentation network Mask-RCNN [37] was employed to select N foreground points from LiDAR points by distinguishing their 2D projection positions. The N points were re-projected onto the image to acquire sparse depth label for calculating the depth loss of foreground pixels. In addition, the foreground loss will be given more weight to balance the contributions of foreground and background pixels.

With the image feature and image depth, the frustum feature $F_{T}\in R^{H_{F}\times W_{F}\times D\times C}$ can be constructed as follows

(1) $$F_{T}=SM\left(D_{bin}\right)⊗F_{I}$$

where ⊗ was the outer product and SM represented the SoftMax function. Equation (1) stated that at each image pixel, the image features were weighted by the depth distribution values along the depth axis. CNN was known to extract image features in convolutional kernels, where object pixels may be surrounded by the pixels of the background or other objects. In contrast, the frustum feature network lifted image features onto depth bins along the depth axis, which enabled the model to discriminate misaligned features in 3D space.

**Frustum-to-point Transition Module.** The submodule aims to extract the PPC features from frustum feature. There are two issues to be addressed regarding the choice of PPC. First, due to the presence of depth errors, the PPCs converted from image may not be consistent with the distribution of the object in space. Second, the number of PPC is proportional to the image resolution, and the number is generally large. Nevertheless, only in the area where the point cloud is relatively sparse can PPC play an important role by compensating for the missing object information.

**Figure 4:**
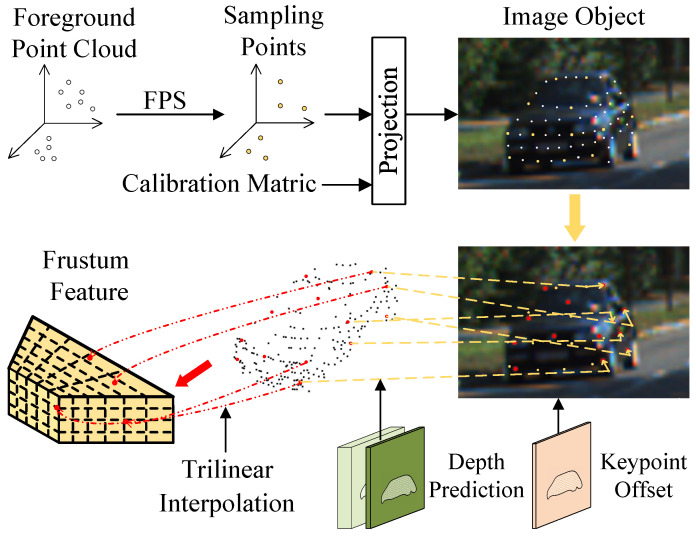
Illustration of Frustum-to-point Transition module. The white point is the foreground point, yellow is sampled foreground point, and red is the keypoint of the object.

For the first issue, we applied the farthest point sampling (FPS) algorithm to select M of the previous N foreground points as the initial PPCs in Figure 4. Foreground points are used because they have more accurate depth values near the projected positions in the image. Accordingly, the projected coordinates $\left\{c_{i}=\left(u_{i},v_{i}\right)|i=1,\ldots ,M\right\}$ of M foreground points can be obtained via calibration matrix.

As for the second issue, the object keypoints that focus on more representative object parts are introduced as the final PPCs. Keypoints are defined as locations that reflect the local geometry of an object, such as points on mirrors and wheels. To determine the locations of keypoints in 3D space, inspired by Deformable Convolutional Networks [41], a 2D keypoint offset was predicted which represented the offset of each pixel on the image to its nearest keypoint. For the M projected coordinates, M keypoint offsets were acquired as follows

(2) $$O_{I}\left(c_{i}\right)=\left(\Delta u_{i},\Delta v_{i}\right)=\sum_{q}G\left(c_{i},q\right)O_{I}\left(q\right)$$

where *q* enumerated the nearby integral locations of $c_{i}$ on the image and G(·,·) was the bilinear interpolation kernel. Keypoint offset $O_{I}\in R^{H_{F}\times W_{F}\times 2}$ was predicted when generating image features illustrated in Figure 3.

Then, the locations of the 2D keypoints can be obtained as $\left\{c_{i}^{{}^{\prime }}=\left(u_{i}+\Delta u_{i},v_{i}+\Delta v_{i}\right)|i=1,\ldots ,M\right\}$ by moving the M pixels according to the corresponding keypoint offsets. With the depth value $depth(c_{i}^{{}^{\prime }})$ of the updated positions, the final PPCs can be determined in camera space. As shown in Figure 4, the features of the PPCs $F_{pseu}\in R^{M\times C}$ can be extracted from the frustum feature $F_{T}$ using the trilinear interpolation. Subsequently, in order to process the PPCs features and LiDAR points features simultaneously, the PPC $p_{i}^{{}^{\prime }}=\left(u_{i}+\Delta u_{i},v_{i}+\Delta v_{i},depth\left(c_{i}^{{}^{\prime }}\right)\right)$ was re-projected to LiDAR space from the camera space by the transformation function $f_{re-proj}$ in KITTI

(3) $$\begin{array}{cc} p_{i}^{pseu} & =\left(x^{{}^{\prime }},y^{{}^{\prime }},z^{{}^{\prime }}\right)=f_{re-proj}\left(p_{i}^{{}^{\prime }}\right)\\ & =T_{LiDAR\leftarrow refer}\cdot T_{refer\leftarrow camera}\cdot p_{i}^{{}^{\prime }} \end{array}$$

where $p_{i}^{pseu}$ was the final coordinate of the *i*th PPC, $T_{refer\leftarrow camera}$ was the transformation matrix from the coordinate of color camera to the reference camera, and $T_{LiDAR\leftarrow refer}$ was the transformation matrix from the reference camera to LiDAR. To verify the effectiveness of Frustum-to-point Transition module, an alternative directly using M initial foreground points as PPCs and extracting their features in the same way was provided. In Section 4, the comparison between two strategies on the KITTI dataset will be presented.

Overall, the multi-modal detection task is transformed into single-modal detection by using PPC instead of image to convey object information. The unified point-based representation helps to make subsequent interactions across multi-modal features easier.

### 3.2. Two-Stream Feature Extraction Network

Multiple multi-modal methods [10,13,15] used a two-stream structure to process image and point cloud separately. Limited by the local receptive field of traditional building blocks, e.g., CNN and sparse convolution, these methods struggled to capture all useful feature relationships. In addition, feature alignment and fusion between image and point cloud were still tricky problems.

Based on the unified point-based representation described above, a two-stream feature extraction network was developed to learn the features of point cloud and image at the point level. The two-stream network was mainly built on a transformer for better feature learning and fusion. It had the inputs of the coordinates of point clouds $P_{raw}\in R^{N\times 3}$ and the coordinates of PPCs $P_{pseu}\in R^{M\times 3}$, and the corresponding features were $F_{raw}\in R^{N\times C_{1}}$ and $F_{pseu}\in R^{M\times C_{2}}$. Here, the feature of raw point $p_{j}$ was represented as $f_{j}^{raw}=\left(v_{j},r_{j},g_{j},b_{j},r_{j}\right)\in R^{C_{1}}$, where $v_{j}$ was a one-hot class vector indicating the confidence score of specific class, $\left(r_{j},g_{j},b_{j}\right)$ was the normalized RGB pixel-values of projected location of $p_{j}$, and $r_{j}$ was the reflectance. The feature channel $C_{2}$ of pseudo point $p_{i}$ was the same as the image feature channel *C*.

**Figure 5:**
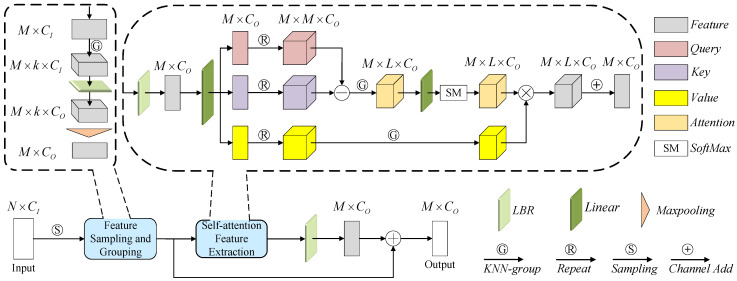
Illustration of PTD. The letters near the box indicate its shape, e.g., N × C, N is the number of points and C is the dimension numbers of features. LBR combines Linear and BatchNorm layers and a ReLU function. KNN-group indicates the grouping operation with k-Nearest Neighbors algorithm.

**Point Transition Down.** In the two-stream network, a stacked PTD encoder was responsible for iteratively extracting multilevel point-based representations. Based on recent attempts [24,25] at object classification, PTD integrated the feature sampling and grouping, self-attention feature extraction and forward-feedback network into a independent module. In Figure 5, PTD first subsampled M points from the input point $P_{I}$ (here $P_{raw}$ or $P_{pseu}$ can act as $P_{I}$) and use *k*-NN algorithm to construct a neighbor embedding for each point. Then, an LBR (Linear layer, BatchNorm layer and ReLU function) operator and a max-pooling operator (MP) were used to encode local features as follows

(4) $$f_{local}\left(p\right)=\mathrm{MP}\left(\mathrm{LBR}\left(concat_{q\in knn(p,P_{I})}\cdot f_{I}\left(q\right)\right)\right)$$

where $f_{I}\left(q\right)$ was the feature of point *q* which belonged to the neighbor of point *p*, $knn(p,p_{I})$ was k-nearest neighbors of point *p* in $P_{I}$.

Next, we sent the local feature $F_{local}\in R^{M\times C_{O}}$ into self-attention feature extraction network to learn long-range dependencies of the features. The relationship between the *query* (*Q*), *key* (*K*), *value* (*V*) matrices and self-attention was as follows

(5) $$\begin{array}{c} \left(Q,K,V\right)=LBR\left(F_{local}\right)\cdot W_{e}\\ Q,K,V\in R^{M\times C_{O}},W_{e}\in R^{C_{O}\times 3C_{O}}\\ Q^{{}^{\prime }}=rg\left(Q\right),K^{{}^{\prime }}=rg\left(K\right),V^{{}^{\prime }}=rg\left(V\right)\\ A^{{}^{\prime }}=SM\left(\alpha \left(Q^{{}^{\prime }}-K^{{}^{\prime }}+\delta \right)\right) \end{array}$$

where $W_{e}$ was the learnable weights of the linear layer and rg(·) represented repeat and grouping operation. $Q^{{}^{\prime }},K^{{}^{\prime }},V^{{}^{\prime }}\in R^{M\times L\times C_{O}}$ were the outputs after repeat and grouping operation related to the input *Q*, *K*, and *V*. Furthermore, a position encoding defined as $\delta =\theta (p_{i}-p_{j})$ was added to the attention, where $p_{i}$, $p_{j}$ were the coordinates of points *i* and *j*. θ and α both consisted of two linear layers and a ReLU function. Thereafter, the output of PTD could be derived as

(6) $$F_{O}=\beta \left(sum\left(A^{{}^{\prime }}\cdot \left(V^{{}^{\prime }}+\delta \right)\right)\right)+F_{local}$$

where sum(·) represented the element-wise product, + denoted channel-wise summation along the neighborhood axis, and β was an LBR operator.

In the point cloud branch, the stacked PTD encoder (including four PTD modules) was used to learn point cloud features. In the PPC branch, the PPTD encoder adopted the same structure to extract image features.

**Point Transition Up.** In the point cloud branch, the stacked PTU decoder aimed to restore the point cloud to its initial number and obtained the multi-scale features for proposal generation. PTU can be easily constructed by replacing the feature sampling and grouping in PTD with the inverse distance-weighted average operation while keeping the other structures intact. The inverse distance-weighted average operation was proposed as the skip connection in PointNet++ [19]

(7) $$f_{int}\left(p_{i}\right)=\frac{\sum_{j=1}^{k}w_{j}\cdot f_{e}\left(p_{j}\right)}{\sum_{j=1}^{k}w_{j}}$$

where $w_{j}=\frac{1}{d{\left(p_{i},p_{j}\right)}^{p}},j=1,\ldots ,k$, $p_{i}$ was the coordinate of the interpolated point, $p_{j}$ was the coordinate of the neighboring point of $p_{i}$, d(·,·) denoted the Euclidean distance between two points, and $f_{int}\left(p_{i}\right)$ denoted the interpolated features of $p_{i}$. Let p=2, k=3 be the same settings in PointNet++ [19], then, the interpolated features were added by the skip connection features as

(8) $$F_{I}^{n}=F_{int}^{n}+\mathrm{LBR}\left(F_{skip}^{n}\right),n=1,2,3,4$$

where $F_{skip}^{n}$ was the *n*-th output of the PTD and $F_{int}^{n}$ was the interpolated feature of the *n*-th PTU. $F_{I}^{n}$ was used as the input of the remaining structure of the *n*-th PTU. On the contrary, in the PPC branch, a stacked FP decoder with four FP layers was used to recover the initial PPCs. Since the position of the PPC was defined on the object keypoint, the distribution of the PPC was more focused on the object surface than the point cloud directly sampled from the LiDAR. Meanwhile, considering the large memory and time overhead of PTU itself, the FP layer was selected to handle the PPC that did not require a large receptive field.

**Figure 6:**
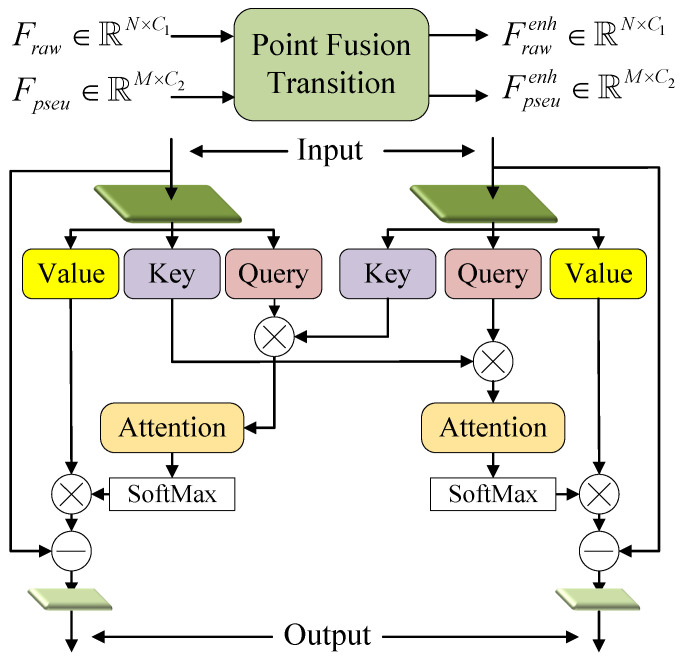
Illustration of PFT. It is employed to perform bi-directional information exchange between two point-based features from different modalities.

**Point Fusion Transition.** According to the above introduction, the stacked PTD encoders of the two branches simultaneously extracted point-based features layer-by-layer. However, the features from the point cloud branch lacked the semantic and textural information about the object, and the features from the PPC branch lacked the geometry information for locating the object. Moreover, both the point cloud provided by LiDAR and the image-generated PPC were inevitably contaminated by noise. To address these problems, a dual input and dual output PFT module was designed for feature fusion in Figure 6. PFT fused two input features based on cross-attention and produced two enhanced features as the inputs to the next level. Finally, an additional PFT was used to fuse the outputs of the two branches (see Figure 2) to obtain the final point representations.

PFT module was also based on transformer and the *Q*, *K*, and *V* matrices were generated separately for the two inputs

(9) $$\begin{array}{c} \left(Q_{raw},K_{raw},V_{raw}\right)=F_{raw}\cdot W_{raw}\\ \left(Q_{pseu},K_{pseu},V_{pseu}\right)=F_{pseu}\cdot W_{pseu}\\ Q_{raw},K_{raw},V_{raw}\in R^{N\times C_{e}}\\ Q_{pseu},K_{pseu},V_{pseu}\in R^{M\times C_{e}}\\ W_{raw}\in R^{C_{1}\times 3C_{e}},W_{pseu}\in R^{C_{2}\times 3C_{e}} \end{array}$$

where $W_{raw}$ and $W_{pseu}$ were both learnable weights. Then, the cross-attention for each data is defined as

(10) $$\begin{array}{c} A_{raw}=SM\left(\sigma \left(K_{raw}\cdot Q_{pseu}^{T}\right)\right)\\ A_{pseu}=SM\left(\varepsilon \left(K_{pseu}\cdot Q_{raw}^{T}\right)\right)\\ A_{raw}\in R^{N\times M},A_{pseu}\in R^{M\times N} \end{array}$$

where σ and ε both comprised two linear layers and a ReLU function. Here, we multiplied the *K* matrix of one modality by the *Q* matrix of the other modality to generate cross-attention. It differed from the way computed in PTD. This practice was inspired by HVPR [42] which took voxel-based features as queries and computed matching probabilities between the voxel-based features and the memory items through dot product. In Section 4, we conducted ablation experiments to compare the effects of different attention calculation ways. Finally, the enhanced features as the outputs of PFT can be expressed as

(11) $$\begin{array}{c} F_{raw}^{enh}=\mathrm{LBR}\left(F_{raw}-\left(A_{raw}\cdot V_{pseu}\right)\right)\\ F_{pseu}^{enh}=\mathrm{LBR}\left(F_{pseu}-\left(A_{pseu}\cdot V_{raw}\right)\right) \end{array}$$

It was worth mentioning that Zhang et al. [34] proposed a similar structure to PFT. However, they had the limitations that the information can only flow from the image domain to the point domain. In contrast, PFT conducted bidirectional information exchange which provided semantic information for point cloud and geometry information for PPC.

### 3.3. RPN and Refinement Network

The two-stream feature extraction network described in Section 3.2 aimed to learn expressive features for every LiDAR point. After that, the features will be sent to the RPN to generate proposals. To obtain high-quality proposals, 3D votes were computed as suggested by ImVoteNet [14], since 3D votes can help narrow down the search space from point to proposal center. The votes were then concatenated with the output of the two-stream network. Finally, the enhanced features were fed into the RPN that included a classification head and a regression head. After acquiring the proposals, non-maximum suppression (NMS) was applied to eliminate redundant proposals. The remaining proposals were sent to the refinement network for generating bounding boxes. In the experiments, two refinement strategies, including point cloud region pooling and RoI-aware point cloud feature pooling were adopted, as proposed by PointRCNN [2] and Part-A2 [43], respectively. Actually, our PTA-Det can be plugged into most point-based detectors as multi-modal detectors.

### 3.4. Overall Loss Function

The model is optimized by a multi-task loss which can be formulated as

(12) $$L_{total}=\lambda_{depth}L_{depth}+\lambda_{rpn}L_{rpn}+\lambda_{rcnn}L_{rcnn}$$

where the $L_{depth}$ denotes the loss of depth prediction for generating the PPCs in Pseudo Point Cloud Generation network. $L_{rpn}$ is the loss of the two-stream feature extraction network to generate the proposal. $L_{rcnn}$ is the loss of the refinement network. $\lambda_{depth}$, $\lambda_{rpn}$, and $\lambda_{rcnn}$ are fixed loss weighting factors. $L_{depth}$ can be computed as

(13) $$L_{depth}=L_{bin}+\lambda_{1}L_{res}$$

where $\lambda_{1}$ is the balance weight for depth residual with the setting of $\lambda_{1}=10$. $L_{bin}$ and $L_{res}$ are defined as

(14) $$\begin{array}{c} L_{bin}=\frac{1}{N}\sum_{i=1}^{N}\mathrm{FL}\left(D_{bin}\left(u_{i},v_{i}\right),D_{gt\_bin}^{i}\right) \end{array}$$

(15) $$\begin{array}{c} L_{res}=\frac{1}{N}\sum_{i=1}^{N}\mathrm{SML}\left(D_{res}\left(u_{i},v_{i}\right),D_{gt\_res}^{i}\right) \end{array}$$

where FL denotes focal loss [44] and SML is Smooth−L1 loss. $D_{gt\_bin}^{i}$ and $D_{gt\_res}^{i}$ denote bin-based index and normalized residual value of the *i*th foreground point’s depth. $D_{bin}\left(u_{i},v_{i}\right)$ and $D_{res}\left(u_{i},v_{i}\right)$ have been introduced in Section 3.1, and the focal loss is adopted in $L_{bin}$ with the setting of α=0.25 and λ=2.0. $L_{rpn}$ consists of a classification loss and a regression loss as

(16) $$L_{rpn}=L_{cls}+\lambda_{2}L_{reg}$$

with

(17) $$L_{cls}=-\alpha_{t}{\left(1-c_{t}\right)}^{\gamma }log(c_{t})$$

and

(18) $$\begin{array}{c} c_{t}=\left\{\begin{array}{cc} c & if\;p\;is\;the\;foreground\;point\;\\ 1-c & otherwise \end{array}\right. \end{array}$$

(19) $$L_{reg}=\sum_{u\in (x,y,z,l,w,h,sin\theta ,cos\theta )}\mathrm{SML}\left({r\hat{es}}_{u},res_{u}\right)$$

where $\lambda_{2}$ is the balance weight, *c* denotes the classification confidence for the point *p*, and $L_{cls}$ is supervised by focal loss. ${r\hat{es}}_{u}$ and $res_{u}$ are the predicted residuals and residual labels of the foreground point. Smooth-L1 loss is used to regress the offsets of the location, size, and direction. The loss of the refinement network is the same as that of PointRCNN [2] or Part-$A^{2}$ [43].

## 4. Experiment

The model was evaluated on KITTI, a commonly used benchmark dataset for 3D object detection. PTA-Det was built on the basis of the OpenPCDet [45] which was an open-source project for 3D object detection.

### 4.1. Dataset and Evaluation Metric

**KITTI Dataset.** The KITTI dataset consists of 7481 training samples and 7518 test samples, focusing on the categories of car, pedestrian and cyclist. Following the investigations [5,7], the original training samples are further separated into a training set (3712 frames) and a validation set (3769 frames). The Average Precision (AP) is calculated using 40 recall positions as the validation metric according to Geiger et al. [4]. All the objects are classified into easy, moderate, and hard levels based on their sizes, occlusion, and truncation. In the experiments, the results on the validation set are reported for all difficulty levels.

**nuScenes Dataset.** The nuScenes dataset is a multi-modal dataset for 3D object detection. It includes 1000 scenes that is composed of 700 scenes for training and 150 scenes for validation. The remaining scenes are used for testing. For each scene, it captures about 20 s of video material with 6 cameras, 1 LiDAR and 5 radars, and is annotated with 3D bounding boxes every 10 frames. According to official evaluation metrics for 3D detection, mean Average Precision (mAP) and nuScenes detection score (NDS) are reported by our model.

### 4.2. Implementation Details

**Network settings.** As a multi-modal 3D object detection method, LiDAR points, RGB image, and camera calibration matrices were taken as inputs. We assumed that the 3D scene was constrained to [(0,70.4), (−40,40), (−3,1)] meters along the X (forward), Y (left), and Z (up) axes in the LiDAR coordinate, respectively. During depth prediction, the depth range was discretized into 80 bins. Unlike LiDAR coordinate, the camera coordinate was set along the X (left), Y (down), and Z (forward) axes. The transformation between two coordinates can be achieved by a calibration matrix. For experiments on nuScenes, we set the 3D scene to [(−51.2, 51.2), (−51.2, 51.2), (−5,3)] meters along the X, Y and Z axes.

For each 3D scene, 16,000 LiDAR points and image with a resolution of 1280 × 384 were used as the initial inputs to the model. In the Pseudo Point Cloud Generation Network, Mask-RCNN implemented by detectron2 [46] was used to generate the foreground mask, and 4096 foreground points were selected through the mask to guide the depth prediction. In a scene where the number of foreground points was less than 4096, the remaining points were randomly selected from the background points. Then, 1600 points were further sampled from the foreground points as the input to the point cloud branch, where the stacked PTD encoder had the point numbers set to 800, 400, 200, 100, respectively. By contrast, the Pseudo Point Cloud Generation Network produced 480 PPCs for the PPC branch, and the PPTD encoder iteratively extracted the features of PPCs, whose numbers were 240, 120, 60, and 30, respectively.

**Training scheme.** After generating proposals in RPN, redundant proposals were eliminated using NMS. The thresholds were set to 0.8 and 0.85 in the training and testing stages. In the refinement network, we utilized the IoU between the proposal and the ground truth to distinguish between positive and negative proposals. Following PointRCNN [2], different thresholds were selected for classification and regression. Specifically, the proposals with IoU scores higher than 0.6 were considered positive samples for classification. In contrast, the proposals with IoU scores lower than 0.45 were considered negative samples. The proposals with IoU scores higher than 0.55 were used to calculate the regression loss.

We trained the model with a batch size of 2 for 80 epochs and adopted the Adaptive Moment Estimator (Adam) optimizer with an initial learning rate, weight decay, and momentum at 0.01, 0.01, and 0.9, respectively. All experiments were conducted on two RTX 3090 GPUs using the deep learning framework PyTorch [47]. It is worth noting we did not use any data augmentation techniques during training.

### 4.3. Main Results

**Results on KITTI dataset.** PTA-Det was compared with several LiDAR-only and multi-modal 3D object detection methods, and the results were summarized in Table 1. Two versions of PTA-Det were given, one of which adopted the point cloud region pooling strategy and the other adopted the RoI-aware point cloud pooling strategy. The mAP of the former was 1.54% and 0.72% higher than that of the latter for the car and pedestrian categories, respectively. However, in the cyclist category, the latter outperformed the former by 1.06%. The results showed that the refinement strategy employed by the former was sufficient to accurately localize the object when image features were used as a complement. Although the latter can better capture the point cloud distribution in the proposals, its advantage was not obvious in our model.

Table 1 showed that PTA-Det exhibited better performance than a variety of previous multimodal methods by about 2% to 14% mAP in the car category. However, the current PTA-Det showed less mAP in the pedestrian and cyclist categories. The reason was that, considering the large memory overhead in the transformer, the number of point clouds input to the model was reduced. This made the points on the surface of small objects more sparse, which in turn led to poor detection performance of the model on small objects.

To reveal the reason for the degradation, we studied the performance of PointRCNN [2] under different sampling strategies, as summarized in Table 2. PointRCNN [2] abstracted a set of point representations using iterative set-abstraction (SA) blocks. In the default case, it sampled 16,384 points from the scene as input, and used four SA layers in sequence to sample the points with group sizes of 4096, 1024, 256, and 64, respectively. Three different sampling strategies were also presented, the last of which used the same number of points as PTA-Det.

As shown in Table 2, the mAP of PointRCNN declined significantly in all categories as the number of input points decreased. Comparing the fourth strategy with PTA-Det-1, it was proved that PTA-Det can reach a higher accuracy than PointRCNN with the same number of points. These investigations supported our conjecture above on the reason for the poor performance of PTA-Det when detecting small objects. Therefore, although Table 1 showed that PTA-Det performed worse than the LiDAR-only method, we believe that our model will achieve more competitive performance as long as a reasonable memory reduction strategy is developed to reduce the memory overhead while increasing the number of point clouds.

**Figure 7:**
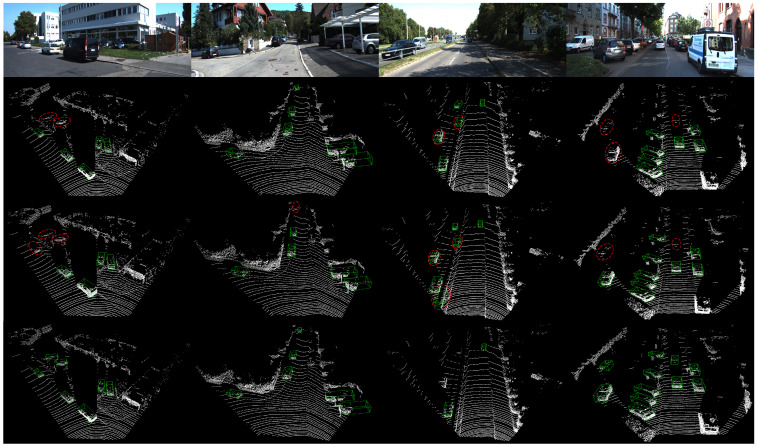
Visual comparisons with two baseline methods. The second row shows the detection results of the modified PointRCNN, the third row shows Part-$A^{2}$ and the fourth row shows ours. The detection results of the first, second, and four scenes show our method can detect distant objects than these LiDAR-only methods under the same point cloud input. From the detection results of the third scene, for the van which is very close to the car structure, PTA-Det can effectively reduce false positive instances due to the addition of image semantic information.

**Figure 8:**
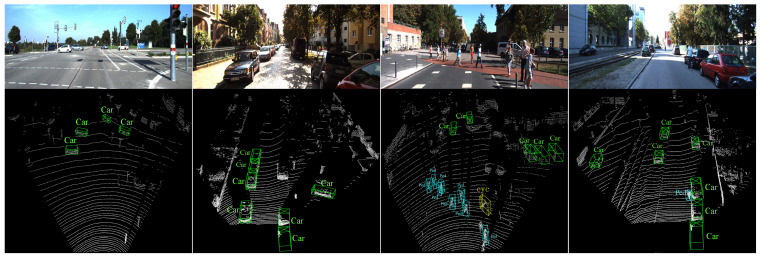
Visualized results by PTA-Det on KITTI test set. Green/turquoise/yellow bounding box indicates car/pedestrian/cyclist category, respectively. The scenes are arranged from left to right. The first and fourth show that PTA-Det performs well against distant objects. The second successfully detects all cars and excludes the object of van category. From the third scene, we observe that PTA-Det performs a little worse with multiple pedestrians and overlooks a car due to occlusion.

In order to further verify the effectiveness of PTA-Det, we provided PointRCNN and Part-$A^{2}$ with the same foreground point input as PTA-Det through the Pseudo Point Cloud Generation Network introduced in Section 3.1, and then compared their detection performances again. The results in Table 3 showed that PTA-Det outperformed the two methods in BEV detection and 3D detection of the car category. In addition, to illustrate the superiority of using pseudo point clouds to represent image features, we have added multiple multimodal detection methods for further comparison, which used the same data processing approach as the other methods. The results in Table 3 showed that PTA-Det still showed competitive results in 3D Detection of car category under the easy and moderate level. At the same time, we also noticed that the performance of PTA-Det was slightly worse than that of the latest multimodal detectors under the BEV Detection and the difficulty level of 3D Detection. The possible reason for this phenomenon was that we only used the feature information near the keypoints of the image to interact with the point cloud features. Compared with other methods extracting the image features in the whole image or 2D ROIs, we may lose some key object information. Then, we showed the comparisons of the detection performance in multiple scene instances in Figure 7 to further prove the advantage of our model in car category. Finally, we made several qualitative investigations to illustrate the effectiveness of PTA-Det on KITTI test set, as visualized in Figure 8.

**Results on nuScenes dataset.** We conducted experiments on the popular nuScenes dataset for 3D object detection to further validate the effectiveness of PTA-Det. We presented detection results of two versions of PTA-Det on the nuScenes validation set. From Table 4, PTA-Det-1 obtained 59.45 mAP and 65.23 NDS, and PTA-Det-2 obtained 57.32 mAP and 63.57 NDS. We also reported detailed detection results for all car-related categories for both methods. Except for the construction vehicle category, PTA-Det-1 surpassed the performance of PTA-Det-2 in all other metrics. Meanwhile, PTA-Det-1 outperformed the previous best baseline CenterPoint [52] by +3.84 mAP and +0.51 NDS, and our model also outperformed many other single- or multi-modal detection methods with fewer input point clouds.

### 4.4. Ablation Studies

We conducted the ablation studies on KITTI dataset to evaluate the influence of each module or strategy on the final results, including PTD, PTU, PFT, two selection strategies for PPC, and the calculation way of attention in PFT. For comparison, a baseline two-stream structure was designed to replace the two-stream network of PTA-Det. It also contained a point cloud branch and a PPC branch, both of which were built with PointNet++ block and FP layer. In addition, the interaction between the two branches was achieved through simple feature concatenation at multiple levels. Other network structures and parameter settings remained unchanged in the baseline model. Both PTA-Det and the baseline selected the RoI-aware point cloud feature pooling strategy in the refinement network. To trade off the speed and accuracy of PTA-Det, we chose ResNet-50 as the image backbone and followed the research of Pan et al. [54] to use the computational cost reduction strategy in PFT.

**Effects of PTD and PTU modules.** From the first three rows in Table 5, the baseline obtained a 43.25% mAP. After replacing all PointNet++ blocks with PTD, the mAP was improved by 5.59%. If all FP layers in the point cloud branch were replaced with PTU, the mAP was improved by 11.13%. When we used the two strategies together in the 5th row, the mAP was improved by 15.52% relative to that of the baseline. The improvements were attributed to the self-attention mechanism, which can aggregate long-distance dependencies better than Pointnet++ [19] blocks and FP layers [19].

**Effects of PFT and Fusion Operation in PFT.** Table 5 also showed that after introducing PFT into the baseline network, although the mAP of the pedestrian category was improved by 0.24%, the performance of the other categories became worse. This was because it was difficult for PFT to directly compute the attention of multi-scale features from SA blocks in Pointnet++. However, as can be found in the 5th and 6th rows, PFT can clearly improve the mAP if accompanied by PTD and PTU. A total improvement up to 16.88% had been realized when PTD, PTU, and PFT were used simultaneously.

To analyze the impact of the structure of the PFT on the results, three schemes had been studied in Table 6. In PFT, by default, the cross-modal features of each modality were subtracted from its input features, and attached it with an LBR as the output. This module was denoted as ${\mathrm{PFT}}_{-}$. We then replaced subtraction with summation and concatenation, denoted as ${\mathrm{PFT}}_{+}$ and ${\mathrm{PFT}}_{c}$, respectively. As in Table 6, ${\mathrm{PFT}}_{-}$ exhibited better performance than the two schemes. The improvements were +3.13% and +1.05% on mAP, showing the advantage of subtraction in PFT.

**Influence of the Calculation Way of Attention in PTD, PTU and PFT.** In PTD and PTU, the subtraction between the query matrix and the key matrix was used to compute the self-attention of the point features, while the multiplication between the two matrices was used in PFT to compute the cross-modal attention. In order to investigate the impact of the two attention computation ways on mAP, we combined the PTD, PTU, and PFT modules using subtraction or multiplication operation to form four different schemes for comparison. Table 7 showed that the fourth way had 4.69% to 7.68% higher mAP than the other three ways. The subtraction between point-based features helped capture the relationship between different features in the same dimension, since it provided the attention computation for each channel. This was crucial for PTD and PTU to obtain intra-modal features. In contrast, inter-modal features varied greatly and a larger perspective was required to capture their relationships. Multiplication can produce a scalar to measure the distance between features across channels. Thus, multiplication was more suitable for computing cross-attention than subtraction for PFT.

**Effect of Sampling Strategy in Pseudo Point Cloud Generation Network.** In Section 3.1, we mentioned two strategies to obtain PPCs: (1) apply FPS algorithm to sample the foreground points and the sampled points were used as the final PPCs, which was denoted as FPS; (2) apply the keypoint sampling strategy based on 2D keypoint offset, which was denoted as KPS. Table 8 showed that KPS performed better. The results showed that using the object keypoints as PPCs can provide more information about the object than PPCs directly sampled from the foreground points.

## 5. Conclusions

In this paper, a method named PTA-Det is proposed, which uses pseudo points as an intermediate modality between the image and the point cloud to solve the multi-modal 3D object detection problem. The pseudo points generated by a Pseudo Point Cloud Generation network not only contain representative semantic and textural information, but also compensate for the missing information of the object. The generated PPC and point cloud are then fed into a two-stream attention-based feature extraction network to learn intra-modal features. Simultaneously, multiple PFT modules in the backbone fuse the two features layer by layer using cross-attention. PTA-Det aims to explore a more reasonable fusion method for camera image and LiDAR points and form a plug-and-play module that can be combined with LiDAR-only methods. Extensive experiments are conducted on the KITTI and nuScenes datasets and competitive results are given. PTA-Det shows better performance on car category than most existing multimodal detection methods on multiple datasets. It is worth mentioning that our method can achieve better accuracy than the LiDAR-only methods under the same number of input points. In short, the experimental results indicate that PTA-Det could be a robust approach for 3D object detection in autonomous driving and many other applications.

## Figures and Tables

**Table 1:** Comparison with popular 3D object detection methods on the KITTI validation split. The available results are evaluated by mAP for each category. ‘L’ and ‘R’ stand for LiDAR and image, respectively. ${}^{★}$ indicates no data augmentation technique is applied. PTA-Det-1 and PTA-Det-2 represent our method adopts the refinement network proposed in PointRCNN and Part-$A^{2}$, respectively. The optimal results are marked in bold font.

Method	Modality	Car				Pedestrian				Cyclist			
		Easy	Mod.	Hard	mAP	Easy	Mod.	Hard	mAP	Easy	Mod.	Hard	mAP
SECOND [20]	L	88.61	78.62	77.22	81.48	56.55	52.98	47.73	52.42	80.58	**67.15**	**63.10**	**70.28**
$\mathrm{PointRCNN}{\;}^{★}$ [2]	L	89.41	78.10	75.51	81.01	**70.39**	**60.41**	**51.48**	**60.79**	**81.81**	58.10	53.86	64.59
Part-$A^{2\;★}$ [43]	L	85.28	74.22	69.85	76.45	53.24	46.80	41.06	45.50	66.51	41.78	39.30	66.54
SE-SSD [48]	L	**90.21**	**86.25**	**79.22**	**85.23**	-	-	-	-	-	-	-	-
MV3D [5]	L+R	71.29	62.68	56.56	63.51	-	-	-	-	-	-	-	-
AVOD-FPN [6]	L+R	84.41	74.44	68.65	75.83	-	58.80	-	-	-	49.70	-	-
F-PointNet [7]	L+R	83.76	70.92	63.65	72.78	**70.00**	**61.32**	53.59	**61.64**	77.15	56.49	53.37	62.34
SIFRNet [49]	L+R	85.62	72.05	64.19	73.95	69.35	60.85	52.95	61.05	80.97	60.34	56.69	65.97
EPNET [13]	L+R	**88.76**	**78.65**	**78.32**	**81.91**	66.74	59.29	**54.82**	60.28	**83.88**	**65.50**	**62.70**	**70.69**
PTA-Det-1	L+R	**86.31**	**77.06**	**70.28**	**77.88**	61.77	51.84	46.98	53.53	70.61	49.02	45.54	55.06
PTA-Det-2	L+R	84.72	74.45	69.86	76.34	60.84	52.48	45.11	52.81	72.43	49.17	46.75	56.12

**Table 2:** Detection performance of PointRCNN on the validation set under different sampling strategies.

Sampling Strategy		3D Object Detection (%)			
**Input Point**	**Group Size**	**Car**	**Cyc.**	**Ped.**	**mAP**
16,384	4096–1024–256–64	81.01	64.59	60.79	68.80
8000	2000–1000–500–250	76.55	48.28	52.86	59.23
4000	2000–1000–500–250	68.65	37.27	45.09	50.34
1600	800–400–200–100	38.28	31.05	2.36	23.90

**Table 3:** Performance comparisons on the KITTI validation set. All methods adopt the same foreground point sampling strategy proposed in Pseudo Point Generation Network, and they do not use any data augmentation technique for a fair comparison. All results are reported by the average precision with 0.7 IoU threshold and 40 recall positions for car category. * indicates our re-implementation. The optimal results are highlighted in bold front.

Method	Modality	BEV Detection (%)			3D Detection (%)		
		Easy	Mod.	Hard	Easy	Mod.	Hard
PointRCNN [2]	L	89.83	81.75	76.89	83.41	72.64	67.87
Part-$A^{2}$ [43]	L	89.22	82.62	78.31	84.62	71.74	68.85
PointPainting [10]	L+R	90.32	82.64	76.91	84.67	74.67	68.87
AutoAlignV2 * [50]	L+R	90.95	86.03	**83.31**	86.27	76.72	70.81
EPNet++ * [15]	L+R	89.46	85.23	80.32	83.21	69.58	68.51
SFD * [51]	L+R	**92.72**	**86.28**	81.73	86.17	74.97	**71.98**
PTA-Det-1	L+R	90.52	84.47	76.39	**86.31**	**77.06**	70.28
PTA-Det-2	L+R	91.33	83.25	78.67	84.72	74.45	69.86

**Table 4:** Comparison of previous baseline works on the nuScenes validation set. None of the methods in the table use data augmentation techniques and all take the same point cloud input as our model. C.V. is the abbreviation of construction vehicle. mAP, NDS scores, and APs are reported for vehicle-related categories. The optimal results are highlighted in bold front.

Method	mAP	NDS	Car	Truck	Bus	Trailer	C.V.
PointPillar [53]	43.53	56.83	77.43	44.65	53.78	47.61	15.76
PointPainting [10]	45.61	54.52	78.62	42.64	52.42	48.64	16.12
SECOND [20]	50.22	61.92	84.29	51.54	53.13	50.32	21.82
CenterPoint [52]	55.61	64.32	84.32	53.74	62.42	54.24	23.62
PTA-Det-1	**59.45**	**65.23**	**86.12**	**55.93**	**66.43**	**57.39**	25.92
PTA-Det-2	57.32	63.38	85.43	53.63	62.38	56.32	**26.43**

**Table 5:** Contributions of PTD/PTU/PFT modules to PTA-Det.

Component				3D Object Detection (%)			
**Baseline**	**PTD**	**PTU**	**PFT**	**Car**	**Cyc.**	**Ped.**	**mAP**
✓				68.94	32.84	27.96	43.25
	✓			65.77	42.62	38.12	48.84
		✓		72.66	43.38	47.11	54.38
			✓	66.74	32.44	28.20	42.46
	✓	✓		75.03	51.73	49.56	58.77
	✓	✓	✓	75.98	52.00	52.41	60.13

**Table 6:** Comparison of fusion structure in PFT to PTA-Det.

Integrating Scheme	3D Object Detection (%)			
	Car	Cyc.	Ped.	mAP
${\mathrm{PFT}}_{c}$	75.22	48.26	47.52	57.00
${\mathrm{PFT}}_{+}$	75.41	51.69	50.13	59.08
${\mathrm{PFT}}_{-}$	75.98	52.00	52.41	60.13

**Table 7:** Comparison of calculation ways of attention. The three consecutive symbols represent the way of attention computation used in PTD, PTU, and PFT, where – stands for subtraction, × for multiplication.

Calculation of Attention	3D Object Detection (%)			
	Car	Cyc.	Ped.	mAP
– – –	72.47	50.26	43.58	55.44
×××	70.98	45.27	43.03	53.09
× × –	69.34	48.22	39.79	52.45
– – ×	75.98	52.00	52.41	60.13

**Table 8:** Comparison of sampling strategies of Pseudo Point Cloud Generation network to PTA-Det.

Sampling Strategy		3D Object Detection (%)			
**FPS**	**KPS**	**Car**	**Cyc.**	**Ped.**	**mAP**
✓		73.83	53.76	48.79	58.79
	✓	75.98	52.00	52.41	60.13

## Data Availability

Publicly available datasets were analyzed in this study. These data can be found here: https://www.cvlibs.net/datasets/kitti (accessed on 7 December 2022) and https://www.nuscenes.org/nuscenes (accessed on 7 December 2022).
